# Real-world treatment patterns and outcomes in hormone receptor–positive, HER2-low metastatic breast cancer, 2018–2023: a retrospective, observational, US cohort study

**DOI:** 10.1007/s10549-026-08029-w

**Published:** 2026-07-20

**Authors:** Erica L. Mayer, Simon M. Collin, Sam Hillman, Luis C. Berrocal-Almanza, Joseph Sparano, Clara Lam

**Affiliations:** 1 Department of Medical Oncology, Dana-Farber Cancer Institute, Boston, MA US; 2 Oncology Outcomes Research, Oncology Business Unit, Evidence Generation to Publications (EG2P), AstraZeneca, Cambridge, UK; 3 Center of Oncology Data Excellence (CODE), Oncology Business Unit, Evidence Generation to Publications (EG2P), AstraZeneca, Cambridge, UK; 4 Division of Hematology and Medical Oncology, Icahn School of Medicine at Mount Sinai, Tisch Cancer Institute, New York, NY US; 5 Breast Franchise, Medical Affairs, Oncology Business Unit, AstraZeneca, Gaithersburg, MD US

**Keywords:** Metastatic breast cancer, Hormone receptor–positive, HER2-low, Real-world evidence

## Abstract

**Purpose:**

To investigate real-world demographics, clinical characteristics, treatment patterns, and outcomes in US patients with ≥ 1 line of therapy (LOT) for hormone receptor–positive (HR+) HER2-low metastatic breast cancer (mBC).

**Methods:**

This retrospective cohort study used a US-based electronic health record-derived de-identified database of patients with mBC diagnosed in 2018–2023. Patient demographics, clinical characteristics at date of mBC diagnosis, treatment patterns, and real-world overall survival (rwOS) and progression-free survival (rwPFS) were analyzed.

**Results:**

Of 2662 patients, 49.4% had recurrent mBC. The median (Q1–Q3) number of LOTs was 2 (1–3). Endocrine therapy (ET) plus a cyclin-dependent kinase 4/6 inhibitor (CDK4/6i) was the most common first-line, second-line, and third-line therapy. In LOT1, 86.2% of patients received ET-containing regimens; consecutive use of ET-containing regimens in subsequent lines constituted 66.0%, 46.0%, 28.3%, and 19.3% of patients in LOT2–5, respectively. Of 830 patients who received chemotherapy, a median (Q1–Q3) of 1 (0–2) prior lines of ET-containing regimens were received. Median (95% CI) rwOS from mBC diagnosis was 42.4 (40.7, 45.0) months; 5-year survival was 36.9%. Median (95% CI) rwPFS decreased from 16.3 (15.2, 17.3) months in LOT1 to 9.1 (8.3, 10.0), 6.2 (5.6, 7.2), 5.3 (4.6, 6.1), and 3.8 (3.3, 4.9) months in LOT2–5, respectively.

**Conclusions:**

Treatment of HR+, HER2-low mBC was characterized by progressive endocrine exhaustion followed by later lines of chemotherapy, with outcomes worsening at each subsequent treatment line. Data highlight the need for new, effective treatment options in this setting.

**Supplementary Information:**

The online version contains supplementary material available at 10.1007/s10549-026-08029-w.

## Introduction

In the US, it is estimated that approximately 320,000 new cases of invasive breast cancer (BC) will be diagnosed in 2026, with BC accounting for approximately 30% of all new cancers in women [1]. Determination of the most appropriate treatment regimens for BC is guided by disease characteristics, including hormone receptor status and human epidermal growth factor receptor 2 (HER2) expression [2, 3]. HER2 expression was previously classified as positive or negative, as determined by immunohistochemistry (IHC) and/or in situ hybridization with or without fluorescence (FISH/ISH) assay results [4]. More recently, HER2-low (defined as an IHC score of 1+, or a score of 2+ combined with a negative FISH/ISH test result [ISH−]) and HER2-ultralow (defined as an IHC score of 0 but with faint membrane staining in > 0% and ≤ 10% of tumor cells) have been recognized as subtypes of HER2-negative BC [5]. Patients in the US who have hormone receptor–positive (HR+) HER2-low or HER2-ultralow unresectable or metastatic breast cancer can benefit from HER2-directed therapy after disease progression on endocrine therapy (ET) in the metastatic setting [6]. More than 50% of patients with metastatic breast cancer (mBC) are reported to have HER2-low tumors [7]. The prevalence of HER2-low tumors in mBC is reported to be higher among patients who have tumors that are hormone receptor–positive (HR+; approximately 65–71%) than in patients whose tumors are hormone receptor–negative (HR−; approximately 37–53%) [8, 9].

The first-line (1L) standard of care in the metastatic setting for HR+ and HER2-negative, or HR+ and HER2-low or -ultralow BC is ET, including aromatase inhibitors or selective estrogen receptor degraders (SERDs), with or without a cyclin-dependent kinase 4/6 inhibitor (CDK4/6i). In patients with disease progression after ET with a CDK4/6i, treatment options include subsequent ET combinations (such as ET with inhibitors of the phosphoinositide 3-kinase [PI3K] / protein kinase B [AKT] / mechanistic target of rapamycin [mTOR] pathway) or, in patients for whom ET-based therapies are no longer clinically viable (such as those with endocrine-resistant tumors), chemotherapy (CT) or antibody-drug conjugates (ADCs; including trastuzumab deruxtecan or sacituzumab govitecan) [10–12]. The optimal treatment sequence after disease progression on CDK4/6i-based therapy in HR+, HER2-low mBC remains to be defined, representing an unmet need in this patient population.

Most previous studies have described characteristics and outcomes in cohorts of patients with HR+, HER2-negative mBC without focusing on the more recently defined HER2-low subgroup, despite guidelines indicating that such patients may benefit from therapies targeting non-overexpressed levels of HER2, of which trastuzumab deruxtecan is currently the only available agent [13]. To better characterize treatment patterns and clinical outcomes in this population, a retrospective analysis was conducted among US patients with HER2-low mBC, describing patient demographic and clinical characteristics, treatment patterns, and clinical outcomes from 2018 to 2023.

## Methods

### Study design

This was a retrospective cohort study evaluating the demographic and clinical characteristics, treatment patterns, and outcomes of patients in the US with diagnosed HR+, HER2-low mBC between January 2018 and February 2023 who had received at least one line of therapy (LOT) for mBC. A LOT was defined as the first eligible drug (i.e., systemic antineoplastic treatment) episode plus other eligible drugs initiated within 28 days of the start of the episode (unless the patient was no longer receiving the first eligible drug, in which case the agents were reported as distinct LOTs), and regimens were named according to the combination of therapies in that line. When adjacent episodes had a small overlap (up to 3 days) between an oral agent and any other drug, dates were adjusted per Flatiron rules so that agents remained distinct lines and were not merged into a single LOT. Patient demographic and clinical characteristics at date of mBC diagnosis were examined. Follow-up data were gathered from the patient’s date of mBC diagnosis until their date of death (if available), other outcome-specific event, last known activity date, or the end of the study period (September 30, 2023).

### Data source

This study used the US-based, electronic health record (EHR)-derived Flatiron Health Research Database (FHRD), which receives data from more than 800 sites of care broadly distributed across the US – primarily community and academic oncology settings (approximately 75% and 25%, respectively) [14]. Patient-level medical record data (structured and unstructured) are processed via technology-enabled abstraction and provide information on patient demographics, diagnosis, treatment, and outcomes [14].

### Study population

Patients were eligible for study inclusion if they had histologically or cytologically documented BC as a primary cancer with evidence of advanced disease and were aged at least 18 years at time of diagnosis. Patients were required to have HR+, HER2-low mBC, with no previous indication of HER2-positive disease. HR+ was defined according to ASCO–College of American Pathologists guidelines [15], and HER2-low was defined as IHC 1+ or IHC 2+/ISH–, with no previous indication of HER2-positive disease (as indicated by biomarker testing). HER2 biomarker test data in Flatiron were abstracted from biomarker reports, pathology addendums, or physician notes when no report was available, and could represent any specimen type. Where patients had serial tests in their records, HER2 status was determined from the test result closest to the diagnosis of mBC. Patients were classified as HER2-low if the closest HER2 test result to the diagnosis of mBC was IHC 1+, or if the closest HER2 test result to the diagnosis of mBC was IHC 2+ and the patient had an ISH− test and did not have an ISH+ test within the study period from January 2018 to February 2023. Patients were required to have received at least one LOT for mBC and were excluded if they had received treatment as part of a clinical trial during the study period or if they had another prior primary cancer or other malignancy (except non-melanoma skin cancers or if treated curatively with no evidence of disease for at least 3 years prior to the time of mBC diagnosis).

### Objectives and endpoints

The primary objective of this study was to assess the treatment patterns of patients with HR+, HER2-low mBC from the first LOT (LOT1) to the fifth LOT (LOT5) after diagnosis of mBC. Secondary objectives included describing demographic and clinical characteristics, and estimating clinical outcomes, including real-world overall survival (rwOS) and real-world progression-free survival (rwPFS). rwOS was defined as the time from mBC diagnosis or start of LOT of interest to the date of death due to any cause, and rwPFS was defined as the time from mBC diagnosis or start of LOT of interest to the date of the earliest progression or death due to any cause.

### Statistical methods

Descriptive statistics were used to summarize treatment patterns and demographic and clinical characteristics; treatment patterns and categorical variables were summarized using frequency counts and percentages, and median and interquartile range were used to summarize continuous variables. Time-to-event outcomes were estimated using the Kaplan-Meier method with 95% confidence intervals (CIs). Patients were censored at the last activity date if there was no event or death due to any cause.

## Results

### Patient demographics and baseline disease characteristics

**Fig. 1:**
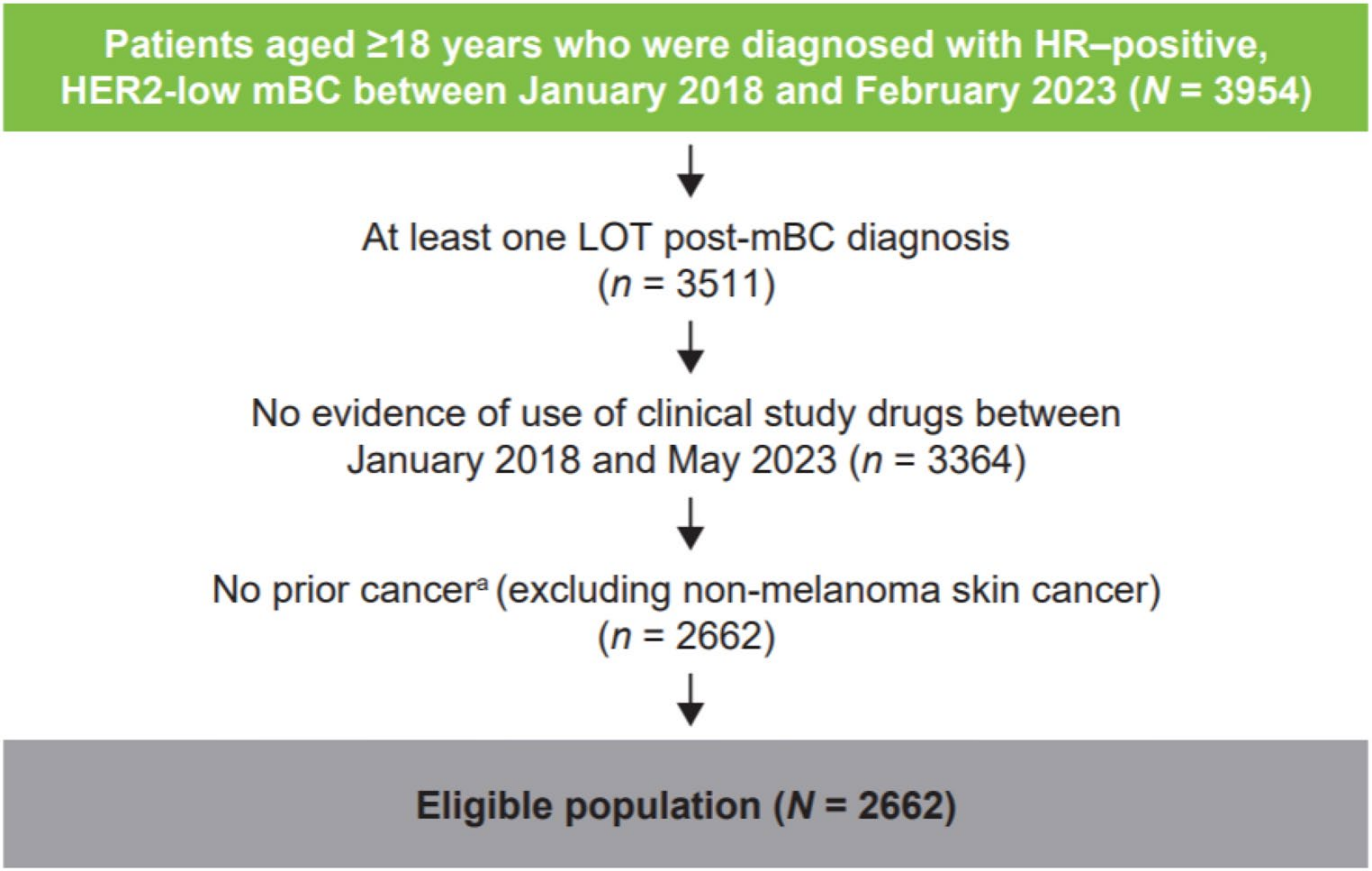
Patient attrition. ^a^Unless curatively treated with no evidence of disease for ≥ 3 years prior to the time of mBC diagnosis. *HER2* human epidermal growth factor receptor 2, *HR* hormone receptor, *LOT* line of therapy, *mBC* metastatic breast cancer

Of 3954 adult patients in the FHRD with HR+, HER2-low mBC diagnosed during the study period (January 2018–February 2023), 2662 met the eligibility criteria for this study (Fig. 1). Patient demographics and clinical characteristics are summarized in Table 1. Patients were mostly female (97.9% [*n* = 2607]), and the median (first quartile [Q1]–third quartile [Q3]) age at mBC diagnosis was 65 (56–74) years. The patient population was predominantly White (61.4%, *n* = 1635), followed by Black (10.4%, *n* = 276), Asian (3.0%, *n* = 79), and Hispanic (0.2%, *n* = 6) patients; 15.3% (*n* = 406) and 9.8% (*n* = 260) of patients were categorized as other and unknown racial groups, respectively. Nearly half of patients (49.4%, *n* = 1316) had recurrent mBC (Stage I/II/III at initial BC diagnosis); 41.2% of patients (*n* = 1098) had de novo mBC (Stage IV at initial diagnosis), and stage at initial diagnosis was not documented for 9.3% of patients (*n* = 248).

### Treatment patterns

Median (Q1–Q3) duration of follow up after diagnosis date was 38.3 (24.4–53.3) months, and the median (Q1–Q3) number of LOTs in the metastatic setting and up to the end of the study was 2 (1–3). At study end, 5.2% of patients (139/2662) at LOT1, 5.4% (79/1459) at the second LOT (LOT2), 4.4% (33/758) at the third LOT (LOT3), 4.5% (18/396) at the fourth LOT (LOT4), and 6.4% (12/187) at LOT5 were still receiving treatment in the corresponding line. Second-line (2L) therapy was received by 1459 patients (54.8%), 758 (28.5%) received third-line (3L), 396 (14.9%) received fourth-line (4L), and 187 (7.0%) received fifth-line (5L) therapy.

**Fig. 2:**
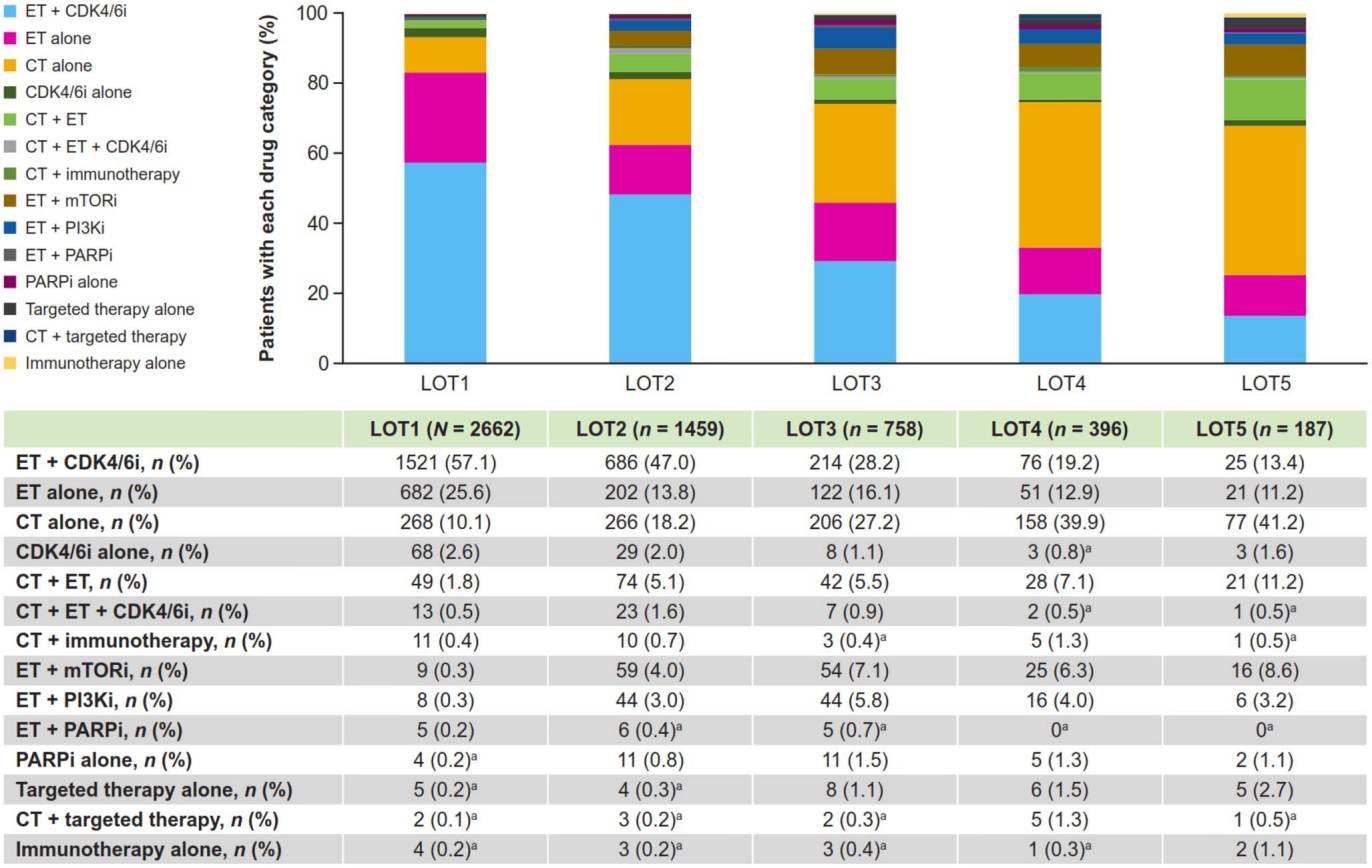
Top ten drug regimens in any line, by LOT. Drug regimens are mutually exclusive. ^a^Not a top ten drug category in the specified LOT. *CDK4/6i* cyclin-dependent kinase 4/6 inhibitor, *CT* chemotherapy, *ET* endocrine therapy, *LOT* line of therapy, *LOT1* first line of therapy, *LOT2* second line of therapy, *LOT3* third line of therapy, *LOT4* fourth line of therapy, *LOT5* fifth line of therapy, *mTORi* mammalian target of rapamycin inhibitor, *PARPi* poly(ADP-ribose) polymerase inhibitor, *PI3Ki* phosphatidylinositol 3-kinase inhibitor

ET-containing regimens accounted for 86.2% of 1L, 76.7% of 2L, 67.2% of 3L, 52.0% of 4L, and 50.3% of 5L treatments, and included monotherapy as well as combinations with CDK4/6i, chemotherapy, targeted therapies (including but not limited to PI3K inhibitors, mTOR inhibitors, and poly[ADP-ribose] polymerase inhibitors), immunotherapy, and/or other agents. The most frequently used regimen in the 1L setting was ET + CDK4/6i, which was received by 57.1% of patients (*n* = 1521), followed by ET monotherapy (25.6%, *n* = 682), CT alone (10.1%, *n* = 268), CDK4/6i alone (2.6%, *n* = 68), and ET + CT (1.8%, *n* = 49). The most frequent regimens in subsequent treatment settings were: LOT2, ET + CDK4/6i (47.0%, *n* = 686); LOT3, ET + CDK4/6i (28.2%, *n* = 214); LOT4, CT alone (39.9%, *n* = 158); and LOT5, CT alone (41.2%, *n* = 77). Of the 2662 patients in the study population, 2113 (79.4%) received a CDK4/6i in any treatment setting, and 511 (19.2%) received a CDK4/6i in at least two treatment settings. The ten most-used drug regimens by treatment setting are shown in Fig. 2. Use of ET + CDK4/6i in LOT1 increased from 51.9% (565/1088) during 2017–2019 to 60.7% (956/1574) during 2020–2023, while use of ET alone decreased from 28.6% (311/1088) to 23.6% (371/1574) and CT alone decreased from 12.1% (132/1088) to 8.6% (136/1574) during these periods.

**Fig. 3:**
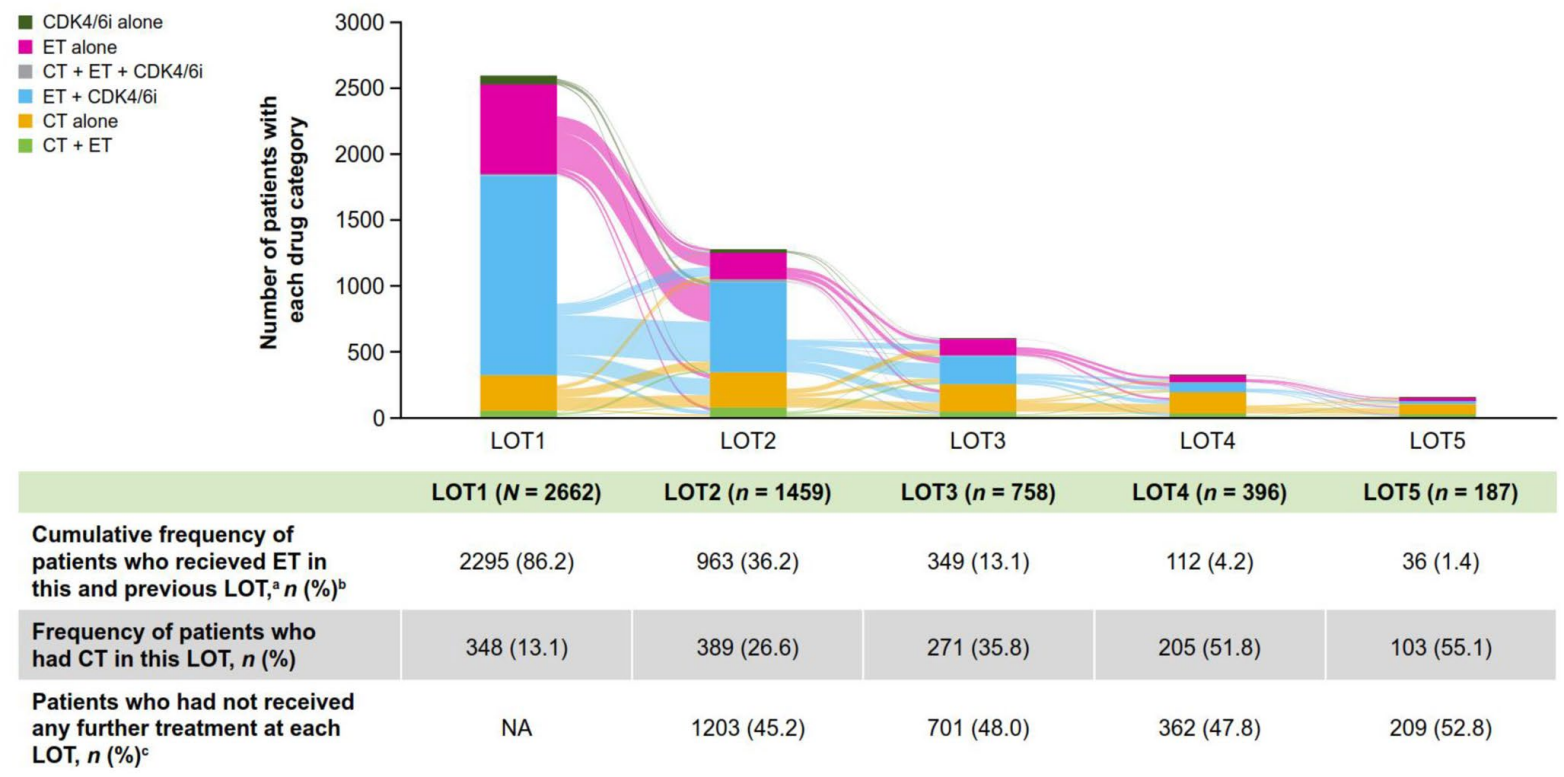
Treatment patterns over LOT. ^a^Only includes patients who received ET in all previous LOTs (other drugs in the LOT are ignored). ^b^Because cumulative data are given, the denominator is 2662. Categories are not mutually exclusive. (some patients may have received ET monotherapy or ET combined with other classes of drugs). ^c^Including patients who did not go on to the next LOT because they were still on a previous LOT at end of study: 1L, 139/2662 (5.2%); 2L, 79/1459 (5.4%); 3L, 33/758 (4.4%); 4L, 18/396 (4.5%); and 5L, 12/187 (6.4%). *1L* first line, *2L* second line, *3L* third line, *4L* fourth line, *5L* fifth line, *CDK4/6i* cyclin-dependent kinase 4/6 inhibitor, *CT* chemotherapy, *ET* endocrine therapy, *LOT* line of therapy, *LOT1* first line of therapy, *LOT2* second line of therapy, *LOT3* third line of therapy, *LOT4* fourth line of therapy, *LOT5* fifth line of therapy, *NA*, not applicable

Of the 2295 patients who received ET-containing regimens in LOT1 (representing 86.2% of patients), 963 went on to receive an ET-containing regimen in LOT2; consecutive ET-containing regimens were received by 349 patients in LOT1–3, 112 in LOT1–4, and 36 in LOT1–5. Consecutive ET-containing regimens represented 66.0%, 46.0%, 28.3%, and 19.3% of treatments received in LOT2–5, respectively. Of the 348 (13.1%) patients who received CT-containing regimens in LOT1, consecutive CT-containing regimens were received by 139 in LOT1–2, 26 in LOT1–3, 4 in LOT1–4, and one patient in LOT1–5, representing 9.5%, 3.4%, 1.0%, and 0.5% of all treatments received in LOT2–5, respectively. Treatment patterns over LOTs are shown in Fig. 3. Of 830 patients who received a CT-containing regimen, 462 (55.7%) had received at least one prior ET-containing regimen; the median (Q1–Q3) number of ET-containing LOTs prior to receiving a CT-containing regimen was 1 (0–2). After treatment with a CT-containing regimen, 329 patients received an ET-containing regimen in at least one later line (representing 12.4% of the overall population, and 39.6% of patients who received CT), comprising 203 and 126 patients who received CT in 1L and 2L+, respectively.

### Clinical outcomes

**Fig. 4:**
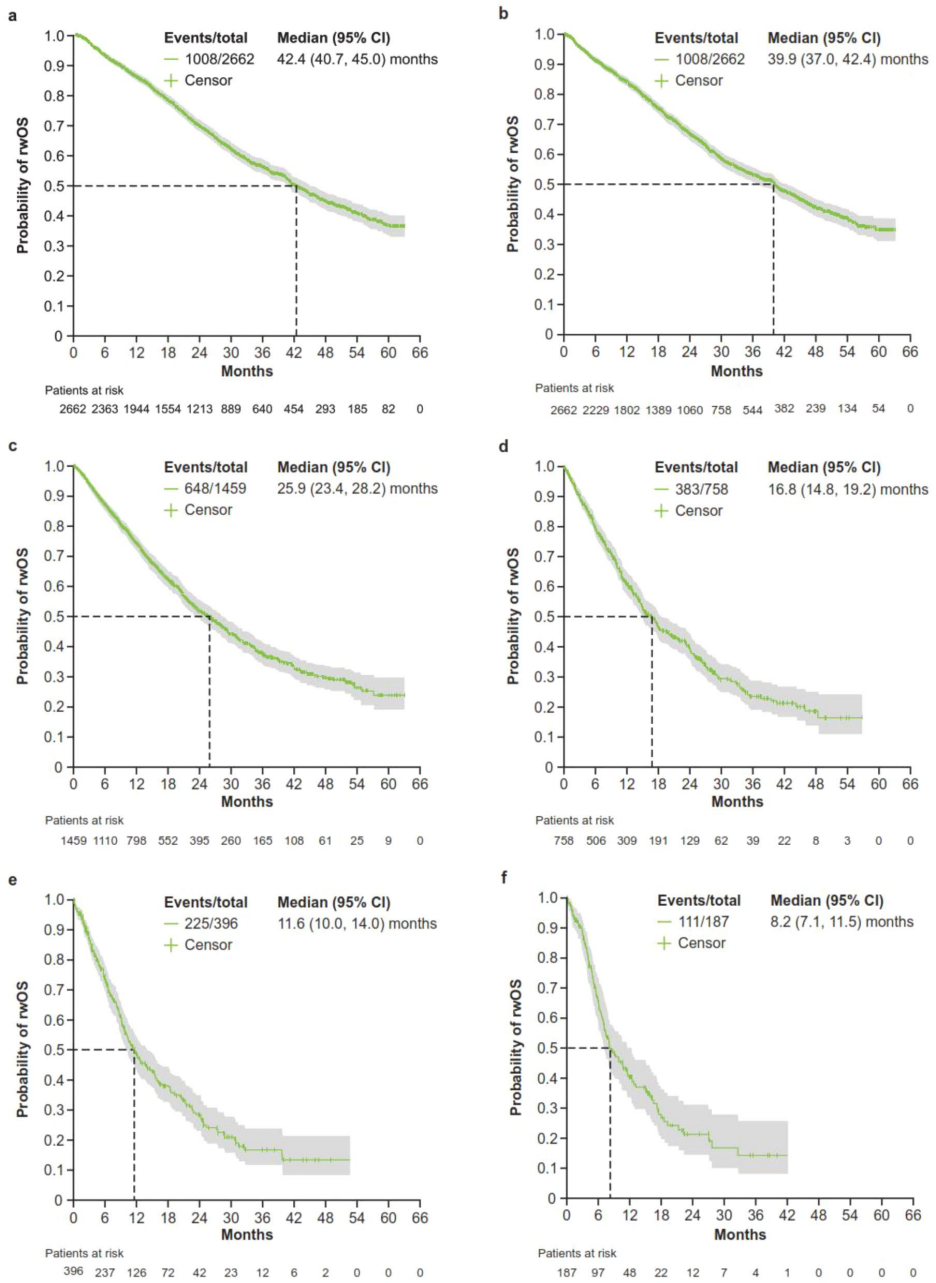
**a–f** rwOS (Kaplan-Meier curve) from **a** mBC diagnosis; **b** LOT1; **c** LOT2; **d** LOT3; **e** LOT4; and **f** LOT5. *CI* confidence interval, *LOT1* first line of therapy, *LOT2* second line of therapy, *LOT3* third line of therapy, *LOT4* fourth line of therapy, *LOT5* fifth line of therapy, *mBC* metastatic breast cancer, *rwOS* real-world overall survival

**Fig. 5:**
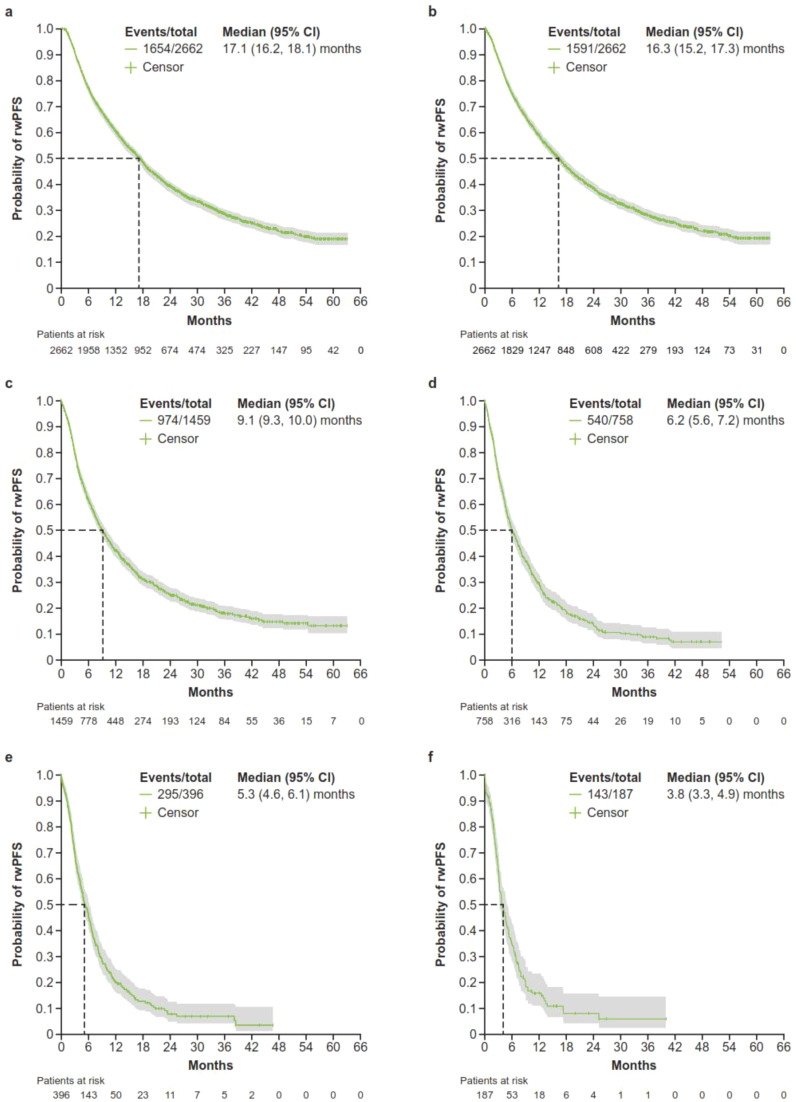
**a–f** rwPFS (Kaplan-Meier curve) from **a** mBC diagnosis; **b** LOT1; **c** LOT2; **d** LOT3; **e** LOT4; and **f** LOT5. *CI* confidence interval, *LOT1* first line of therapy, *LOT2* second line of therapy, *LOT3* third line of therapy, *LOT4* fourth line of therapy, *LOT5* fifth line of therapy, *mBC* metastatic breast cancer, *rwPFS* real-world progression-free survival

The median (95% CI) rwOS from mBC diagnosis was 42.4 (40.7, 45.0) months, decreasing from 39.9 (37.0, 42.4) months at LOT1 initiation to 8.2 (7.1, 11.5) months at LOT5 initiation. rwOS from mBC diagnosis and at initiation of LOT1–5 is shown in Fig. 4. Overall, 36.9% of patients were alive 5 years after mBC diagnosis (Online Resource 1). The median (95% CI) rwPFS from mBC diagnosis was 17.1 (16.2, 18.1) months, decreasing from 16.3 (15.2, 17.3) months at LOT1 initiation to 3.8 (3.3, 4.9) months at LOT5 initiation; rwPFS from mBC diagnosis and at initiation of LOT1–5 is shown in Fig. 5.

## Discussion

This study evaluated treatment patterns and real-world outcomes using secondary EHR data for patients in the US with HR+, HER2-low mBC, from diagnosis of mBC through to LOT5 during the study period of January 2018–February 2023. Most patients (86.2%, *n* = 2295) received ET in the 1L setting either as a monotherapy or in combination with other treatments, most commonly with a CDK4/6i (66.3% of those who received ET, *n =* 1521), consistent with guideline recommendations [10]. Treatment was heterogeneous in subsequent settings, characterized by use of ET with or without CDK4/6i, and CT in later lines. These findings demonstrate increasing therapeutic attrition in HR+/HER2-low disease, with exhaustion of multiple ET and CT options, and diminishing effectiveness (rwPFS) occurring across consecutive LOTs. More than 40% of patients who received CT had not received a prior ET-containing regimen in an earlier setting, despite guidelines recommending sequential ET-based strategies prior to use of CT, except in the event of endocrine resistance or life-threatening visceral disease [16, 17]. Early initiation of CT may occur owing to clinically aggressive disease biology or urgent treatment need in routine practice; however, information on visceral crisis, endocrine resistance, symptomatic burden, and performance status was not available in the database. In addition, despite receiving ET- and CT-based regimens, less than 40% of patients were alive 5 years after mBC diagnosis. Taken together, these results highlight the unmet need for more durable and effective treatment options to improve outcomes in patients with HR+, HER2-low mBC.

Although limited real-world studies in the US have investigated treatment patterns and clinical outcomes in HER2-low mBC, findings previously reported are consistent with the results of our study. A recent analysis of patients in the FHRD with HER2-low mBC who received CT and a subsequent LOT demonstrated that 61.7% of those with HR+ disease received ET in the metastatic setting prior to, or in combination with, the first use of CT [18]. For patients with HR+ mBC, the median time from mBC diagnosis to immediate LOT after first receipt of CT (known as the index LOT) was 22.7 months, and median rwOS from the index LOT was 17.6 months. A similar analysis (also using the FHRD) of patients with HR+, HER2-low mBC who received ET and CT reported that 26.0% of those who had received ≥ 2 lines of ET, CT, and an additional LOT (*N* = 607) subsequently received an additional line of ET ± targeted therapy, further illustrating the treatment pattern heterogeneity in later lines that was observed in our study [19]. A retrospective cohort study of treatment patterns in patients with HER2-low mBC treated in a single US center reported a median overall survival from initiation of 1L treatment of 37.7 months [20], which is comparable to the 39.9 months in our study. Median progression-free survival (PFS) from 1L treatment was 18.0 months, decreasing to 8.1 months from 2L treatment [20], versus 16.3 months and 9.1 months in our study, respectively. Studies in the HER2-negative mBC population are more widely reported than those specifically focusing on the HER2-low subgroup, and findings are generally aligned with those of our study. A retrospective US database analysis of patients with HR+, HER2-negative mBC from 2002 to 2012 that investigated the use of ET showed that, among the 26% (*n* = 3021) of patients who received more than one line of ET, the duration of each subsequent line was shorter than the previous one [21]. Although these data may not reflect recent changes in treatment patterns, these findings align with the observation of ET exhaustion in our study. An analysis from 2010 to 2019, investigating the impact of approval of CDK4/6is, demonstrated that use of ET monotherapy in LOT1 decreased from 2015 onwards, while use of ET + CDK/46i increased in the same timespan [22]. This is consistent with the trends observed in our analysis, where use of ET + CDK4/6i in LOT1 was greater in 2020–2023 compared with 2017–2019, while use of ET monotherapy decreased.

The classification of the HER2-low subset of HER2-negative mBC was largely a result of the efficacy of trastuzumab deruxtecan in such patients [13, 23]. In August 2022, trastuzumab deruxtecan was approved for HR+ and HR−, HER2-low mBC after prior CT in the metastatic setting or following disease recurrence during or within 6 months of completing adjuvant CT [24]. In January 2025, the indication was broadened to allow use after progression following one or more ETs in the metastatic setting [25]. Although HER2-low breast tumors have a higher-than-normal level of HER2, their genomic profile is similar to HER2-negative breast tumors [26], and recent approvals in the HER2-negative mBC setting apply to HER2-low disease. In February 2023, sacituzumab govitecan-hziy was approved for patients with HR+, HER2-negative mBC following ET and at least two additional systemic therapies in the metastatic setting [27]. Although the approvals of trastuzumab deruxtecan and sacituzumab govitecan occurred during the study period, there was likely a delay in adoption, resulting in a lack of representation in the current data. Datopotamab deruxtecan, another ADC, was also approved in January 2025 for HR+, HER2-negative mBC, following ET and CT [28]. The low use of ADCs during the study period may limit the applicability of the findings to the contemporary treatment landscape, which is an important consideration when interpreting them.

Various ETs and targeted therapies have also received regulatory approvals for HER2-negative disease, including the SERDs elacestrant, approved in January 2023 [29] (used in a small proportion of patients in this study), and imlunestrant, which was approved in 2025 [30]. As more therapies targeting actionable tumor mutations are approved, genomic profiling of tumors will grow increasingly relevant to the understanding of whether patients can benefit from tailored treatment strategies. For example, capivasertib and inavolisib both represent recently approved targeted therapies that were not administered to patients included in this study [31, 32]. Further, new agents and indications are being investigated, such as gedatolisib, a multi-target PI3K/AKT/mTOR pathway inhibitor [33], and the SERD giredestrant [34]. Collectively, the array of recent approvals and ongoing investigations demonstrate the rapid advances in individualized care that have already changed, and will continue to contribute to, the rapidly evolving mBC treatment landscape. This is an important consideration when interpreting the findings of the current study.

The main strength of this study is the data source. Flatiron data are abstracted mainly from community-based oncology centers, with approximately 75% of practices in Flatiron being community based rather than academic. Our findings therefore reflect treatment patterns for the majority of patients in the US with HR+, HER2-low mBC [35]. This study had some limitations; namely, the database did not reflect treatment(s) potentially provided in hospitals or other oncology clinics that may not be part of an EHR, leading to potential misclassification or incomplete information. Additionally, the reasons for patients not proceeding to a subsequent LOT were not captured in the database, other than still receiving current LOT at study end; although characterizing reasons for attrition was not an objective of this study, such details would have been beneficial to contextualize our findings. The database also lacks information on clinically relevant variables, such as visceral disease burden, endocrine sensitivity or resistance status, *ESR1*/*PIK3CA* alterations, and CNS involvement. Measuring rwPFS with physician assessments, as opposed to Response Evaluation Criteria in Solid Tumours, limits comparisons with PFS from clinical trial populations. Given the heterogeneity of treatments within LOTs, our estimates of rwPFS by LOT reflect a mix of treatment regimens. As mentioned above, although data were analyzed over a 5-year period up to September 2023, the rapid evolution of treatment patterns is such that these findings may no longer precisely reflect current standards of care. Furthermore, the lack of exploratory subgroup analyses according to, for example, de novo versus recurrent metastatic disease, first-line ET versus CT, HER2 IHC 1+ versus IHC 2+/ISH− disease, and prior CDK4/6 inhibitor exposure limits interpretation of the treatment patterns and outcomes described herein. Finally, although we summarized numbers of patients receiving consecutive lines of endocrine-based and CT-based regimens in each LOT, we did not characterize transitions between these regimens, which might be useful to interpret longitudinal treatment sequencing.

## Conclusions

Although ET with or without CDK4/6i was widely used as a 1L treatment for HR+, HER2-low mBC in the US during the study period (2018–2023), treatment heterogeneity increased in subsequent LOTs, with progressive endocrine exhaustion and increased use of CT alone or in combination with ET and/or CDK4/6i. Outcomes (rwPFS) worsened with each LOT, and fewer than two in five patients survived beyond 5 years, highlighting the need for new treatment options to improve clinical outcomes in this patient population.

**Table 1:** Patient demographics and clinical characteristics

	Overall *N* = 2662
Median age (Q1–Q3) at mBC diagnosis, years	65 (56–74)
Age group at mBC diagnosis, *n* (%)	
< 40 years old	117 (4.4)
40–60 years old	757 (28.4)
> 60 years old	1788 (67.2)
Female sex, *n* (%)	2607 (97.9)
Race, *n* (%)	
White	1635 (61.4)
Black	276 (10.4)
Asian	79 (3.0)
Hispanic	6 (0.2)
Other	260 (9.8)
Unknown	406 (15.3)
Stage at initial BC diagnosis, *n* (%)	
I/II/III (recurrent mBC at study start)	1316 (49.4)
IV (de novo mBC)	1098 (41.2)
Not documented	248 (9.3)
Year of mBC diagnosis, *n* (%)	
2018	524 (19.7)
2019	564 (21.2)
2020	539 (20.2)
2021	522 (19.6)
2022	437 (16.4)
2023	76 (2.9)
Median (Q1–Q3) duration of follow up, months	38.3 (24.4–53.3)

*BC* breast cancer, *mBC* metastatic breast cancer, *Q1* first quartile, *Q3* third quartile

## Supplementary Information

Below is the link to the electronic supplementary material.

Supplementary Material

## Data Availability

The data that support the findings of this study were originated by and are the property of Flatiron Health, Inc. Requests for data sharing by license or by permission for the specific purpose of replicating results in this manuscript can be submitted to PublicationsDataAccess@flatiron.com.

## Abbreviations

1L: First-line
2L: Second-line
3L: Third-line
4L: Fourth-line
5L: Fifth-line
ADC: Antibody-drug conjugate
AKT: Protein kinase B
ASCO: American Society of Clinical Oncology
BC: Breast cancer
CDK4/6i: Cyclin-dependent kinase 4/6 inhibitor
CI: Confidence interval
CT: Chemotherapy
EHR: Electronic health record
ET: Endocrine therapy
FDA: US Food and Drug Administration
FHRD: Flatiron Health Research Database
FISH: In situ hybridization with fluorescence
HER2: Human epidermal growth factor receptor 2
HR^+^: Hormone receptor–positive
HR^−^: Hormone receptor–negative
IHC: Immunohistochemistry
ISH: In situ hybridization
LOT: Line of therapy
LOT1: First line of therapy
LOT2: Second line of therapy
LOT3: Third line of therapy
LOT4: Fourth line of therapy
LOT5: Fifth line of therapy
mBC: Metastatic breast cancer
mTOR: Mechanistic target of rapamycin
mTORi: Mechanistic target of rapamycin inhibitor
PARPi: poly(ADP-ribose) polymerase inhibitor
PFS: Progression-free survival
PI3K: Phosphoinositide 3-kinase
Q1: First quartile
Q3: Third quartile
rwOS: Real-world overall survival
rwPFS: Real-world progression-free survival
SERD: Selective estrogen receptor degrader
