# Supplementary material for: Real-world treatment patterns and outcomes in hormone receptor–positive, HER2-low metastatic breast cancer, 2018–2023: a retrospective, observational, US cohort study
Source: Breast Cancer Res Treat. 2026 Jul 20;218(2):15. doi: 10.1007/s10549-026-08029-w (PMC13385082; doi:10.1007/s10549-026-08029-w)
Supplement: Supplementary file 1 — Supplementary Material [file 10549_2026_8029_MOESM1_ESM.pdf]

Supplementary information

Real-world treatment patterns and outcomes in hormone receptor–positive, HER2-low metastatic breast cancer, 2018–2023: a retrospective, observational, US cohort study

Breast Cancer Research and Treatment

Erica L. Mayer • Simon M. Collin • Sam Hillman • Luis C. Berrocal-Almanza • Joseph Sparano • Clara Lam

Corresponding author:

Simon M Collin

Oncology Outcomes Research, Oncology Business Unit Medical, Evidence Generation to Publications (EG2P), AstraZeneca, Cambridge, UK

simon.collin@astrazeneca.com

Online Resource 1 rwOS probabilities by year since mBC diagnosis

	**Probability of rwOS, % (95% CI)**
1 year	86.0 (84.6, 87.3)
2 years	69.9 (68.0, 71.9)
3 years	56.3 (54.0, 58.7)
4 years	45.0 (42.3, 47.8)
5 years	36.9 (33.6, 40.4)

CI confidence interval, *mBC* metastatic breast cancer, *rwOS* real-world overall survival
